# Kinetics of Ordering and Deformation in Photosensitive Azobenzene LC Networks

**DOI:** 10.3390/polym10050531

**Published:** 2018-05-15

**Authors:** Vladimir Toshchevikov, Tatiana Petrova, Marina Saphiannikova

**Affiliations:** 1 Leibniz-Institut für Polymerforschung, Hohe Str. 6, 01069 Dresden, Germany; toshchevikov@ipfdd.de (V.T.) to_87@bk.ru (T.P.); 2 Institute of Macromolecular Compounds, Russian Academy of Sciences, Bolshoi pr. 31, Saint-Petersburg 199004, Russia; 3 Cherepovets State University, pr. Lunacharskogo 5, Cherepovets 162600, Russia

**Keywords:** photodeformable polymers, polymer networks, liquid crystals, statistical physics, kinetics

## Abstract

Azobenzene-containing polymer networks are unique compounds that are able to change their shape in response to light, which makes them prospective materials for photocontrollable nano-templates, sensors, microrobots, artificial muscles, etc. In present work, we study the kinetics of light-induced ordering and deformation in two-component polymer networks containing optically inert liquid crystalline (LC) mesogens and azobenzene chromophores. By this, we generalize our previous theory [J. Phys. Chem. Lett. 2017, 8, 1094–1098] devoted to the kinetics of photoizomerization in one-component azo-polymers without mesogenic inclusions. The kinetic equations of photoisomerization are used, taking into account the angular selectivity of the photoisomerization with respect to the polarization direction of the light **E**. After multiple trans-cis-trans photoisomerization cycles, the azobenzenes are reoriented preferably perpendicular to the vector **E**. This changes the ordering of the mesogens due to the orientational LC interactions between the components. The light-induced reordering is accompanied by network deformation. Time evolution of ordering and deformation is found as a function of the intensity of light and structural parameters of the LC azo-networks, which define the viscosity, the strength of the LC interactions between the components, the volume fraction of the azobenzene moieties, and the angular distribution of azobenzenes in polymer chains. Established structure-property relationships are in agreement with a number of experimental data.

## 1. Introduction

Liquid crystalline (LC) polymer materials are well-known for their versatility when it concerns a stimuli-triggered mechanical response. As a first, a thermomechanical response in mono-domain liquid crystalline elastomers (LCE) produced by a two-step crosslinking technique [1] was reported by the group of Finkelmann [2]. The preferential orientation of liquid crystalline moieties (mesogens) was imposed by stretching after the first crosslinking step and then fixed in the second crosslinking step [1]. Such uniaxially aligned mono-domain samples exhibit a substantial thermally induced contraction along the orientation direction of mesogens (nematic director) upon the nematic-isotropic phase transition [2,3,4]. The effect is reversible and was explained by a strong elastic coupling between the LC ordering and the conformations of network strands [5,6,7].

Over the years, surface alignment methods, mostly rubbing and photoalignment, have emerged as a key technology for the generation of spatially varying complex director fields inside the LCE samples. Especially attractive is a photoalignment technique based on the ability of azobenzene chromophores to orient perpendicular to the electric field vector of the linearly polarized laser light. This technique was originally proposed to align small LC molecules in display and optical applications [8,9,10]. It has significant advantages compared to the usual rubbing of the alignment surfaces being a non-contact method with a high resolution. Both surfaces of a liquid crystalline cell can be spin coated by azobenzene-based photoalignment material. Optical patterning of the alignment layer can be performed nowadays with an extremely high resolution as described for example in Ref. [11]. There, to imprint complex director fields, the laser beam has been collimated to a spot as small as 100 μm × 100 μm and moved controllably along the surface of alignment layer. Inscription occurs very rapidly and precisely due to a high writing intensity, ~40 W/cm^2^, of the collimated beam, whose polarization is controlled optically at each spot. When the alignment cell is filled with the mixture of monomers, the photopatterned surfaces of the cell dictate a local orientation of the nematic director, which propagates through the cell thickness. In such a way, not only splay alignments but also sophisticated topological defects [9,10] can be imprinted, which then dictate a complex thermomechanical response of the LCE samples, for example conical actuation upon heating [11,12].

Even more fascinating is a photomechanical response of similar LCEs containing azobenzene chromophores in their side and main chains or simply as a dopant [13,14]. For example, a reversible elongation-contraction [15] as well as bending-unbending behavior [16,17] was observed in mono-domain samples under alternating illumination with ultraviolet and visible light. The driving force for the large shape changes arises from a variation in alignment order of the liquid-crystalline groups caused by the *trans-cis* photoisomerization of the azobenzene chromophores [18]. The effect is known as the dilution of LC order by non-mesogenic cis-isomers due to their bent form. Complex director fields can be also imprinted into azo-containing LCEs. This leads to sophisticated photomechanical responses: reversible twisting motions in LC springs [19], three-dimensional (3D) fingerprints activated in azo-containing cholesteric LC networks by the UV light [20], a blue light-driven artificial flytrap based on the splay-aligned LCE actuator [21], and caterpillar-like crawling and wave-like movements [22]. Some of these motions resemble autonomous mechanical actions in living systems [21,22].

To induce a noticeable photomechanical response in azo-containing LC networks, not only the effect of LC dilution upon illumination with the UV light but also strong reorientation effects under the linearly polarized visible light have been successfully utilized [23,24,25]. For example, the direction of photoinduced bending of the polymer network with azobenzene LC moieties can be reversed by switching the polarization of the laser beam in orthogonal directions [23]. This was explained by reorientation of azobenzene LC moieties perpendicular to the beam polarization resulting in expansion/contraction of the polymer network along the polarization direction. The bending of the film is caused by attenuation of visible light across the film thickness due to a strong absorption by azobenzene moieties. Interestingly, even azobenzene-functionalized polyimides with high modulus, ~3 GPa, demonstrate large photoinduced bending induced by linearly polarized blue light of moderate intensity [26,27]. For a recent review on photomechanical effects in LC materials see the relevant contributions in [28].

Previous theoretical works to explain photomechanical effects in LC networks considered the dynamics of the trans-cis isomerization in the presence of UV light [29,30,31,32]. Hence, only the effect of LC dilution by bent cis-isomers was taken into account. The angular selectivity of trans-cis isomerization in azobenzene-containing elastomers was first considered in the early papers from our group [33,34,35]. There, we used an effective orientation potential to describe reorientation of chromophores perpendicular to the electric field vector after multiple trans-cis-trans photo-isomerization cycles. It was predicted that, depending on the chemical structure, amorphous azobenzene elastomers either expand or contract along the polarization direction of visible light due to reorientation of the chromophores. The effects of LC interaction between the azobenzenes on the static ordering and photodeformation were studied in Refs. [36,37], where a light-induced transition from a uniaxial to the biaxial state has been predicted. The theory was then generalized for the case of a two-component polymer network containing both the azobenzenes and optically inert mesogens [38,39]. Application of the orientation potential to describe photomechanical properties of azobenzene-containing materials of different structures was discussed in detail in Ref. [40].

The effects of bent cis-isomers were accounted for in the study [39], which allowed us to consider both the reorientation and dilution effects induced in the azobenzene LC elastomers by the polarized laser beam. The strength of each effect depends on the light wavelength and can be properly accounted for when the relative fractions of trans- and cis-isomers are known as a function of time at any wavelength in the absorption region of the azobenzenes. For that, we solved explicitly the kinetic equations of photoisomerization in the presence of linearly polarized light of constant intensity [41,42]. Up to now, the most elaborated theoretical framework has been developed by us to study transient light-induced ordering and deformation in azobenzene-containing materials as a function of the strength of LC interactions, the light wavelength, and material viscosity [42].

In the present paper, we consider the photoinduced dynamics in a two-component LC network possessing a realistic chemical structure, that is, a structure containing optically inert mesogens and trans- and cis-isomers of the azobenzenes. The theory generalizes our previous studies [41,42] devoted to the kinetics of ordering and deformation in one-component azobenzene-containing polymer networks without mesogenic inclusions. A very rich time-dependent behavior of ordering as represented by LC order parameters and of network deformation has been found for the two-component azobenzene-containing LC polymer networks in agreement with multiple experiments.

## 2. Model and Main Equations

**Figure 1:**
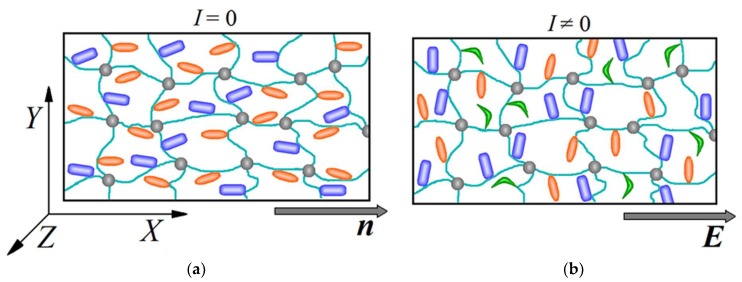
Schematic representation of a two-component polymer network containing the azobenzenes (orange ellipsoids) and mesogens (blue moieties) in side chains at the absence (**a**) and presence (**b**) of polarized light. The green moieties in Figure (**b**) depict the bent cis-isomers of the azobenzenes.

An azobenzene-containing LC polymer network is modelled as an ensemble of polymer chains (network strands) between network junctions (see Figure 1). Each network strand is built from N freely jointed rigid (Kuhn) segments; $N_{A}$ segments contain the azobenzenes in side chains, $N_{M}$ segments bear optically inert mesogens in side chains, and $\left(N-N_{A}-N_{M}\right)$ segments contain neither azobenzenes nor mesogens. The orientation distributions of azobenzenes and mesogens in the side chains are defined by the distribution functions $W_{A}\left(\alpha_{A}\right)$ and $W_{M}\left(\alpha_{M}\right)$, where $\alpha_{A}$ and $\alpha_{M}$ are the angles formed by the long axes of azobenzenes and mesogens with respect to the long axis of the rigid chain segment.

The introduced parameters N, $N_{A}$, and $N_{M}$ as well as the distribution functions $W_{A}\left(\alpha_{A}\right)$ and $W_{M}\left(\alpha_{M}\right)$ are defined by the chemical structure of the azobenzene LC network. The parameter N is related to the degree of cross-linking of a polymer network; $N_{A}$ and $N_{M}$ define the ratio of azobenzenes and mesogens in a two-component polymer network. The distribution functions $W_{A}\left(\alpha_{A}\right)$ and $W_{M}\left(\alpha_{M}\right)$ are determined by the chemical structure and by the length of spacers. As we showed in our previous works [33,34,35,36,37,38,39,40,41,42], the photomechanical behavior of azobenzene networks of different structures is very sensitive to the orientation distribution of the azobenzenes in side chains.

In the present work, we study the photomechanical properties of the two-component polymer networks which are in the LC state at the absence of light with preferable orientation of the mesogens and azobenzenes along the LC director, **n**, Figure 1a. We consider a geometry in which the electric field vector of the linearly polarized light **E** is applied along the LC-director: **E**||**n**, see Figure 1b. As we have shown in Ref. [37], this geometry provides the largest degree of light-induced deformation.

At the absence of light, all azobenzenes are in the ground trans-state and represent rod-like particles (orange ellipsoids in Figure 1a). Illumination with the polarized light leads to the photoisomerization of the azobenzenes from trans- to bent cis-state (green moieties in Figure 1b). The number of cis-isomers is defined by the balance between the trans-cis photoisomerization and possible back cis-trans transformation and depends on the wavelength of the light. In the UV region, the probability of the back cis-trans photoisomerization is rather low and a large fraction of azobenzenes, ~80%, can be transformed into the cis-state [43]. On the other hand, under illumination with visible light, the back cis-trans photoisomerization process is very intensive and most azobenzenes are in the trans-state [43].

An appearance of the cis-isomers can destroy the LC state due to the dilution of the LC system consisting of rod-like particles (mesogens and trans-isomers of azobenzenes) by the bent cis-isomers. This effect can change the orientation order of both components. Moreover, due to the angular selectivity of the photoisomerization process with respect to the electric field vector **E**, the trans-isomers are reoriented preferably perpendicular to the polarization direction of the light (see Figure 1b). Thus, the dilution effect and the angular selectivity of photoisomerization result in the change of orientation order of the LC azobenzene network.

### 2.1. Light-Induced Deformation of an Azobenzene-Containing LC Network

Reorientation of azobenzenes and mesogens with respect to the polarization direction changes conformations of the network strands due to chemical coupling of the azobenzenes and mesogens with the polymer network. This leads to the network deformation. For polymer networks built from long chains (N>10), the statistics of network strands can be well-described by the Gaussian approximation [7,44,45,46]. In this approximation, the elongation ratio, *λ*, of the network along the polarization direction can be calculated as [7]:

(1) $$\lambda ={\left(\frac{{\langle b_{y}^{2}\rangle }_{0}\cdot \langle b_{x}^{2}\rangle }{\langle b_{y}^{2}\rangle \cdot {\langle b_{x}^{2}\rangle }_{0}}\right)}^{1/3}$$

Here, we introduce the Cartesian coordinate system *XYZ*, in which the *x*-axis is directed along the polarization vector **E** and the two other axes lie in the plane perpendicular to the vector **E** (see Figure 1). In Equation (1), $\langle b_{x}^{2}\rangle {}_{0}$ and $\langle b_{y}^{2}\rangle {}_{0}$ are the mean-square projections of the network strands on the *x*- and *y*-axes, respectively, at the absence of light; and $\langle b_{x}^{2}\rangle$ and $\langle b_{y}^{2}\rangle$ are the mean-square projections of the network strands in a deformed network under illumination. Each network strand is built from rigid segments containing the azobenzenes and mesogens in side chains and, thus, the mean-square projection of the network strand on the *β*-axis (*β* = *x*, *y*, *z*) can be written as:

(2) $$\langle b_{\beta }^{2}\rangle =N_{A}\langle l_{A,\beta }^{2}\rangle +N_{M}\langle l_{M,\beta }^{2}\rangle +\left(N-N_{A}-N_{M}\right)\cdot (l^{2}/3)$$

where $\langle l_{A,\beta }^{2}\rangle$ and $\langle l_{M,\beta }^{2}\rangle$ are the projections of the rigid segments bearing azobenzenes and mesogens, respectively, on the *β*-axis. We assume that the network strands are not LC objects and the orientation distribution of $\left(N-N_{A}-N_{M}\right)$ chain segments bearing neither mesogens nor azobenzenes is isotropic. Therefore, their mean-square projections are equal to $l^{2}/3$, where *l* is the length of the chain segment. At the absence of light, the mean-square projections of the network strands can be calculated similarly to Equation (2):

(3) $${\langle b_{\beta }^{2}\rangle }_{0}=N_{A}{\langle l_{A,\beta }^{2}\rangle }_{0}+N_{M}{\langle l_{M,\beta }^{2}\rangle }_{0}+\left(N-N_{A}-N_{M}\right)\cdot (l^{2}/3)$$

where ${\langle \ldots \rangle }_{0}$ means averaging at the absence of light. Substituting Equations (2) and (3) into Equation (1), one can see that the network deformation depends on the number fractions $N_{A}/N$ and $N_{M}/N$.

The orientation distribution of chain segments can be related to the orientation distributions of the azobenzenes and mesogens. To obtain these relationships, we use the distribution functions $W_{A}\left(\alpha_{A}\right)$ and $W_{M}\left(\alpha_{M}\right)$ for the polar angles $\alpha_{A}$ and $\alpha_{M}$. The azimuthal orientation of the chain segments around the azobenzenes and mesogens is assumed to be random since the network strands are not LC objects in our model. Averaging over the orientation of chain segments around the long axes of azobenzenes and mesogens, we obtain:

(4) $$\langle l_{i,x}^{2}\rangle =\frac{1+2S_{i}\cdot q_{i}}{3}\cdot l^{2}{,}^{}\langle l_{i,y}^{2}\rangle =\langle l_{i,z}^{2}\rangle =\frac{1-S_{i}\cdot q_{i}}{3}\cdot l^{2}.$$

Here, $S_{i}$ is the order parameter of azobenzenes (i=A) and mesogens (i=M):

(5) $$S_{i}=\frac{3{\langle \cos^{2}\theta \rangle }_{i}-1}{2}$$

where θ is the angle between the principal axis of an azobenzene (or mesogen) and the x-axis. The order parameter is known to change between minimal and maximal values: S∈[−0.5, 1]. The maximal and minimal values, S=1 and S=−0.5, correspond to a fully oriented ensemble of rod-like particles along the *x*-axis or perpendicular to it, respectively. Here, we note that the order parameter $S_{A}$ for azobenzenes includes averaging over all trans- and cis-isomers because all isomers influence the chain conformations and deformation of the network.

Equation (4) contains the structural parameter $q_{i}$, which defines the orientation of the azobenzenes (i=A) and mesogens (i=M) with respect to the main chain:

(6) $$q_{i}=\frac{3\langle \cos^{2}\alpha_{i}\rangle -1}{2}$$

Here, averaging is performed with respect to the distribution functions $W_{i}\left(\alpha_{i}\right)$ introduced above. Similarly to the order parameter S, the structural parameter q takes its value in the range q∈[−0.5, 1]. The minimal value q=−0.5 corresponds to the chemical structures with a preferable orientation of the azobenzenes and the mesogens perpendicular to the main chains, whereas the maximal value q=1  corresponds to the structures with a preferable orientation of the azobenzenes and the mesogens parallel to the main chains.

Thus, Equations (1)–(6) relate the deformation of the network with its structural parameters $N_{A,M}/N$ and $q_{A,M}$ as well with the order parameters of the azobenzenes and mesogenes $S_{A,M}$, which define the reorientation of the azobenzenes and mesogenes under illumination with light. To determine the time evolution of the order parameters for the azobenzenes and mesogens, we use the kinetic equations of photoisomerization.

### 2.2. Kinetic Equations of Photoisomerization

The photoisomerization process is angular dependent: the maximal/minimal probability of the trans-cis photoisomerization corresponds to the parallel/perpendicular orientation of the trans-isomer with respect to the polarization vector **E**. This effect is known in the literature as an “angular hole burning” or Weigert effect [47,48]. The angular dependence of the probability of the trans-cis photoisomerization can be described in a first approximation by the function $\propto \cos^{2}\theta$ [48,49]. On the other hand, the back cis-trans photoisomerization is angular-independent in a good approximation due to the isotropic polarizability tensor of bent cis-isomers [50]. The mesogens are assumed to be optically inert. Taking into account the assumptions given above, the time evolution of the angular distribution functions $n_{T}\left(\theta \right)$, $n_{C}\left(\theta \right)$, and $n_{M}\left(\theta \right)$ for the trans- and cis-isomers of the azobenzenes and for the mesogens, respectively, can be described by the following kinetic equations, cf. with [41,42,48]:

(7) $$\{\begin{array}{l} \frac{\partial n_{T}(\theta )}{\partial t}=-P_{T}\cos^{2}\theta^{}n_{T}+P_{C}\int d{\Omega^{\prime }}^{}n_{C}(\Omega^{\prime })f_{CT}(\Omega^{\prime }\to \Omega )+{\left(\frac{\partial n_{T}}{\partial t}\right)}_{diff}\\ \frac{\partial n_{C}(\theta )}{\partial t}=P_{T}\int d\Omega^{\prime }\cos^{2}{\theta^{\prime }}^{}n_{T}(\Omega^{\prime })f_{TC}(\Omega^{\prime }\to \Omega )-P_{C}n_{C}+{\left(\frac{\partial n_{C}}{\partial t}\right)}_{diff}\\ \frac{\partial n_{M}(\theta )}{\partial t}={\left(\frac{\partial n_{M}}{\partial t}\right)}_{diff}. \end{array}$$

Here, $P_{T}=k_{TC}I$ and $P_{C}=k_{CT}I+\gamma$ are the probabilities of optical excitations from trans- to cis-isomers and from cis- to trans-isomers, respectively, which are proportional to the intensity of the light, I; $k_{TC}$ and $k_{CT}$ are the rate constants [43]; and the parameter γ corresponds to the thermal relaxation from the excited cis-state to the ground trans-state. Varying the ratio $P_{C}/P_{T}$, the photoisomerization kinetics can be studied in a wide range of wavelengths from UV light ($P_{C}/P_{T}<1$) to visible light ($P_{C}/P_{T}>1$).

Functions $f_{CT,TC}\left(\Omega^{\prime }\to \Omega \right)$ in Equation (7) define the probability of reorientation of the chromophore from an initial state with the angles $\Omega^{\prime }=\left(\theta \prime ,\varphi \prime \right)$ to the new state with Ω=(θ,φ) during the photoisomerization event. Here, φ is the angle between the projection of the chromophore on the *yz*-plane and the *y*-axis; it determines the azimuthal orientation of the chromophore around the polarization vector **E**. Integration in Equation (7) is performed over all orientations of the azobenzenes: dΩ=sinθ dθ dφ, θ∈[0,π], and φ∈[0,2π]. It is assumed that the probability functions $f_{CT,TC}\left(\Omega^{\prime }\to \Omega \right)$ are isotropic with respect to the azimuthal reorientation of the azobenzenes around its initial direction and are defined by the angle *χ* between the initial and final orientations: $f_{CT,TC}=f_{CT,TC}(\chi )$. The redistribution functions $f_{CT,TC}\left(\Omega^{\prime }\to \Omega \right)$ satisfy the normalization condition:

(8) $$\int d\Omega_{}f_{TC,CT}(\Omega^{\prime }\to \Omega )=1$$

which means that the probability of the chromophore reorientation from the orientational state $\Omega^{\prime }$ to some other orientational state Ω on a unit sphere equals unity.

The last terms ${\left(\partial n_{i}/\partial t\right)}_{diff}$ in the three equations of the system (7) describe the rotational diffusion of the orienting moieties: trans- and cis-isomers of the azobenzenes and the mesogens. These terms can be written as follows [51]:

(9) $${\left(\frac{\partial n_{i}}{\partial t}\right)}_{diff}=D_{i}\frac{1}{\sin\theta }\frac{\partial }{\partial \theta }\left[\sin\theta \left(\frac{\partial n_{i}}{\partial \theta }+\frac{n_{i}}{kT}\frac{\partial U_{i}}{\partial \theta }\right)\right]{,}^{}i=T,C,M$$

Here, $D_{i}$ are the rotational diffusion coefficients for the trans- and cis-isomers of azobenzenes as well as for the mesogens included into a polymer network, T is the absolute temperature, k is the Boltzmann constant, and $U_{i}$ are the potentials acting on the orienting moieties due to the LC interactions. It is assumed that the bent cis-isomers are non-LC objects and, thus:

(10) $$U_{C}=0$$

The rod-like trans-isomers and the LC mesogens are influenced by the LC interactions. In the framework of the mean-field approach for a mixture of two nematics [52,53,54,55], the potentials $U_{T,M}$ are defined by the second Legendre polynomial $P_{2}\left(\cos\theta \right)=\left(3\cos^{2}\theta -1\right)/2$:

(11) $$\frac{U_{T}}{kT}=-\left(a_{TT}\Phi_{T}S_{T}+a_{TM}\Phi_{M}S_{M}\right)P_{2}\left(\cos\theta \right)$$

(12) $$\frac{U_{M}}{kT}=-\left(a_{MM}\Phi_{M}S_{M}+a_{TM}\Phi_{T}S_{T}\right)P_{2}\left(\cos\theta \right)$$

Here, the prefactors before $P_{2}\left(\cos\theta \right)$ are the strengths of the self-consistent fields acting on the trans-isomers of azobenzenes and mesogens. They are linear functions of the order parameters of the components, which are related to the distribution functions $n_{T,C,M}\left(\theta \right)$ as follows:

(13) $$S_{i}=\frac{\int d\Omega^{}n_{i}{\left(\Omega \right)}^{}P_{2}(\cos\theta )}{\int d\Omega^{}n_{i}(\Omega )}{,}^{}i=T,C,M$$

Furthermore, the strengths of the self-consistent fields are proportional to the amounts of the trans-isomers of the azobenzenes and mesogens in the azobenzene-containing LC network. We divide the polymer network into two components: (i) the subsystem of the volume fraction $\Phi_{A}$ containing only the azobenzenes; and (ii) the LC network of the volume fraction $\Phi_{M}=1-\Phi_{A}$ containing the network strands and the LC mesogens. The volume fractions of the trans- and cis-isomers are given by:

(14) $$\Phi_{T}=\Phi_{A}\int d\Omega n_{T}(\Omega )\;\mathrm{and}\;\Phi_{C}=\Phi_{A}\int d\Omega n_{C}(\Omega )$$

where the distribution functions $n_{T,C}\left(\theta \right)$ are determined from Equation (7). Integrating the three equations in the system (7) with respect to Ω and using the normalization condition (8) one can find that the distribution functions $n_{T,C,M}\left(\theta \right)$ satisfy the normalization conditions.

(15) $$\int d\Omega \left(n_{T}(\Omega )+n_{C}(\Omega )\right)=1$$

(16) $$\;\int d\Omega n_{M}(\Omega )=1$$

Thus, the kinetic Equation (7) satisfy the condition that the total numbers of azobenzenes and mesogens remain constant.

The coefficients $a_{ij}$ (i,j=T,M) in Equations (11) and (12) are the dimensionless strengths of LC interactions between the rod-like moieties: between the trans-isomers of azobenzenes ($a_{TT}$), between the mesogens in a LC polymer network ($a_{MM}$), and between the trans-isomers of azobenzenes and the mesogens ($a_{TM}$). Note that the coefficients $a_{ij}$ depend on temperature: $a_{ij}=u_{ij}/kT$, where $u_{ij}$ are characteristic energies of the LC interactions, which are determined by the anisotropic van der Waals forces (dipolar and London dispersion forces) as well as by the steric repulsions between the anisotropic particles [53,56]. The characteristic energies $u_{ij}$ are defined by the chemical structure of the anisotropic particles and by their geometrical characteristics, such as their lengths and aspect ratios.

It is well-known from the classical Maier-Saupe theory [53] that, depending on the strengths of the LC interactions $a_{ij}$ as compared to the critical value $a_{c}\approx 4.542$, the system of anisotropic particles can be either in an isotropic ground state with $S_{i}=0$ (if $a_{ij}<a_{c}$) or in an anisotropic LC ground state with $S_{i}>0$ (if $a_{ij}>a_{c}$). The kinetics of the photomechanical behavior of azobenzene-containing materials at weak LC interactions ($a_{ij}<a_{c}$) were considered in detail in our recent work [41]. In ref. [42], the kinetics of light-induced deformation of one-component polymer networks both at weak and strong LC interactions were studied. In the present study, we focus on the photomechanical behavior of two-component azobenzene-containing polymer networks with strong LC interactions at $a_{ij}>a_{c}$.

Kinetics of photoisomerization and light-induced reordering in a two-component LC polymer network are determined by complicated integro-differential Equations (7)–(14) with respect to the distribution functions $n_{T,C,M}\left(\theta ,t\right)$ for all components. One can see that the ordering of orienting moieties of each type (trans- or cis-isomers of azobenzenes and mesogens) is influenced by the ordering of orienting moieties of other types. To simplify the problem, we use the closure approximation proposed earlier for one-component azobenzene-containing polymer networks [42].

### 2.3. Closure Approximation

The complicated task of finding the angular distribution functions $n_{T,C,M}\left(\theta ,t\right)$ can be reduced to a simpler task of solving the kinetic equations for the 2nd and 4th moments of these distributions. For that, we multiply both sides of the Equations (7) by the factor $P_{2}\left(\cos\theta \right)$ and after the integration with respect to Ω obtain:

(17) $$\begin{array}{l} \frac{\partial (\Phi_{T}S_{T})}{\partial t}=-P_{T}\Phi_{T}\left[\frac{3}{2}{\langle \cos^{4}\theta \rangle }_{T}-\frac{1}{2}{\langle \cos^{2}\theta \rangle }_{T}\right]+P_{C}\Phi_{C}S_{C}S_{\chi }^{\left(CT\right)}-6D_{T}\Phi_{T}S_{T}+\\ +9D_{T}\Phi_{T}{\left(a_{TT}\Phi_{T}S_{T}+a_{TM}\Phi_{M}S_{M}\right)}^{}\left[{\langle \cos^{2}\theta \rangle }_{T}-{\langle \cos^{4}\theta \rangle }_{T}\right] \end{array}$$

(18) $$\frac{\partial (\Phi_{C}S_{C})}{\partial t}=P_{T}\Phi_{T}S_{\chi }^{\left(TC\right)}\left[\frac{3}{2}{\langle \cos^{4}\theta \rangle }_{T}-\frac{1}{2}{\langle \cos^{2}\theta \rangle }_{T}\right]-P_{C}\Phi_{C}S_{C}-6D_{C}\Phi_{C}S_{C}$$

(19) $$\frac{\partial S_{M}}{\partial t}=9D_{M}{\left(a_{MM}\Phi_{M}S_{M}+a_{TM}\Phi_{T}S_{T}\right)}^{}\left[{\langle \cos^{2}\theta \rangle }_{M}-{\langle \cos^{4}\theta \rangle }_{M}\right]-6D_{M}S_{M}$$

Here, $S_{\chi }^{\left(TC,CT\right)}=\int^{}d\Omega \;P_{2}\left(\cos\chi \right)f_{TC,CT}(\chi )$ are related to the 2nd moments of the redistribution functions $f_{TC,CT}(\chi )$. The brackets ${\langle \ldots \rangle }_{T,M}$ in Equations (17)–(19) mean the averaging over the subsystems of trans-isomers and mesogens. Furthermore, integrating the first and second equations of the system (7) with respect to Ω, we have:

(20) $$\frac{\partial \Phi_{T}}{\partial t}=-P_{T}\Phi_{T}\frac{2S_{T}+1}{3}+P_{C}\Phi_{C}$$

(21) $$\Phi_{T}+\Phi_{C}=\Phi_{A}$$

One can see that the system of five Equations (17)–(21) contains seven independent variables: $\Phi_{T,C}$, $S_{T,C}$, $S_{M}$, and two 4th moments ${\langle \cos^{4}\theta \rangle }_{T,M}$. Note that the quantities ${\langle \cos^{2}\theta \rangle }_{T,M}$ in Equations (17)–(19) are not independent as they are related to the order parameters ${\langle \cos^{2}\theta \rangle }_{T,M}=\left(1+2S_{T,M}\right)/3$. To close the system of five Equations (17)–(21), we relate ${\langle \cos^{4}\theta \rangle }_{T,M}$ with $S_{T,M}$ using the closure approximation proposed by us in refs [41,42], in which the distribution functions $n_{T,M}\left(\theta ,t\right)$ are approximated as follows:

(22) $$n_{T,M}(\theta )=C_{T,M}\exp[m_{T,M}\cos^{2}\theta ]$$

where $C_{T,M}$ are the normalization constants satisfying conditions (14) and (15). In fact, the closure (22) assumes that at any time the light-induced reordering and effects of the LC interactions are equivalent to the action of some effective potential, $-m_{T,M}\cos^{2}\theta$, where $m_{T,M}$ is the strength of the potential. It was shown in ref. [42] that for LC polymer networks, the proposed closure provides a very good approach since the factor $P_{2}\left(\cos\theta \right)\propto \cos^{2}\theta$ in Equations (11) and (12) for the energy of LC interactions gives a significant contribution to the term ~$\cos^{2}\theta$ for $n_{T,M}\left(\theta ,t\right)$ in Equation (22).

**Figure 2:**
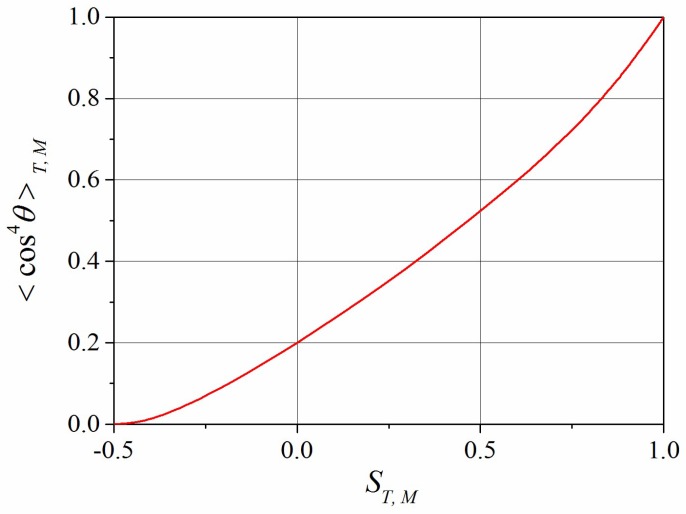
Dependence of ${\langle \cos^{4}\theta \rangle }_{T,M}$ on $S_{T,M}$ in the framework of the closure approximation (22).

Using the closure (22), the order parameters $S_{T,M}$ and the 4th moments ${\langle \cos^{4}\theta \rangle }_{T,M}$ can be calculated simultaneously at any values of $m_{T,M}$. Thus, varying the parameter $m_{T,M}\in \left(-\infty ,+\infty \right)$, we obtain the dependence of ${\langle \cos^{4}\theta \rangle }_{T,M}$ on $S_{T,M}$ as presented in Figure 2. Obviously, to obtain this dependence, we do not need to know explicitly the time dependences $m_{T,M}\left(t\right)$ in Equation (22). Now, having the relationship between ${\langle \cos^{4}\theta \rangle }_{T,M}$ and $S_{T,M}$, the system of five Equations (17)–(21) is expressed in terms of five independent variables: $\Phi_{T,C}$, $S_{T,C}$, and $S_{M}$. We solve this system of equations numerically. Note that using the closure (22), we simplified the problem significantly since we reduced the complete calculation of the angular distribution functions $n_{T,C,M}\left(\theta ,t\right)$ from the set of integro-differential Equation (7) to five scalar parameters. Below, we discuss the results of the numerical calculations.

## 3. Results

The photoisomerization kinetics is studied starting from the initial (dark) state, in which all azobenzenes are in the ground trans-state: $\Phi_{T}\left(t=0\right)=\Phi_{A}$ and $\Phi_{C}\left(t=0\right)=0$. The initial state can be analyzed from the kinetic Equations (17)–(21). Using integration by parts in Equation (13), we obtain the following relationship between the quantities ${\langle \cos^{4}\theta \rangle }_{T,M}$ and ${\langle \cos^{2}\theta \rangle }_{T,M}$ in the framework of the closure approximation (22):

(23) $${\langle \cos^{2}\theta \rangle }_{T,M}-{\langle \cos^{4}\theta \rangle }_{T,M}=S_{T,M}/m_{T,M}$$

Substituting Equation (23) into the kinetic Equations (17) and (19), we obtain that the order parameters in the initial state with $(\partial S_{T,M}/\partial t)=0$ and with $P_{T}=P_{C}=0$ satisfy the following equations:

(24) $$\{\begin{array}{l} m_{T}(S_{T})=\frac{3}{2}\left(a_{TT}\Phi_{T}S_{T}+a_{TM}\Phi_{M}S_{M}\right)\\ m_{M}(S_{M})=\frac{3}{2}\left(a_{MM}\Phi_{M}S_{M}+a_{TM}\Phi_{T}S_{T}\right) \end{array}$$

This is a well-known condition of the self-consistency for a mixture of two nematics [52,53,54,55], i.e., for the rod-like trans-isomers of azobenzenes and mesogens in our case. The condition of self-consistency provides the values of the order parameters $S_{T,M}$ in the global minimum of free energy at $a_{ij}>a_{c}$ [52,53,54,55]. Thus, the closure approximation (22) reproduces exactly the initial state corresponding to the self-consistency condition. The dependences $m_{T,M}\left(S_{T,M}\right)$ in the left-hand sides of Equation (24) are determined from Equation (13), where $n_{T,M}\left(\theta \right)$ are given by Equation (22). Equation (24) represents a system of two equations with respect to two variables $S_{T,M}$ and can be solved numerically. At $a_{ij}>a_{c}$ ($a_{c}\approx 4.542$), this system has a single solution with $S_{T,M}>0$, which corresponds to the global minimum of the free energy.

At t=0, the light is switched on. Initially, all parameters in the right-hand sides of Equations (17)–(20) are known from the self-consistency condition (24) and the time derivatives $\left(\partial S_{T,C,M}/\partial t\right)$ and $\left({\partial \Phi }_{T,C}/\partial t\right)$ in the left-hand sides of Equations (17)–(20) can be calculated. Knowing these time derivatives, we obtain the small increments $\Delta S_{T,C,M}$ and ${\Delta \Phi }_{T,C}$ during the time step Δt and calculate the values $S_{T,C,M}+\Delta S_{T,C,M}$ and $\Phi_{T,C}+{\Delta \Phi }_{T,C}$ corresponding to the next time step. Repeating this cycle many times, we calculate the time dependences $S_{T,C,M}\left(t\right)$ and $\Phi_{T,C}\left(t\right)$ numerically.

Since $\Phi_{C}=0$ at t=0, one can see from Equations (17)–(21) that kinetics of photoisomerization at short times is determined by the characteristic time $\tau_{T}=1/P_{T}\propto 1/I$. Therefore, an increase of the intensity of light I accelerates the kinetics of light-induced ordering and deformation. Dividing both sides of Equations (17)–(21) by the factor $P_{T}$, the kinetic equations can be rewritten in the form containing the dimensionless time $t/\tau_{T}$ and dimensionless quantities ${\tilde{P}}_{C}=P_{C}/P_{T}$, ${\tilde{D}}_{T,C,M}=D_{T,C,M}/P_{T}$, $a_{ij}$, $\Phi_{A}$, and $\Phi_{M}=1-\Phi_{A}$ as parameters. Below, we discuss the kinetics of light-induced ordering and deformation of azo-containing LC networks depending on their optical and physical characteristics in terms of these dimensionless parameters.

### 3.1. Influence of the Amount of Azobenzene Chromophores

**Figure 3:**
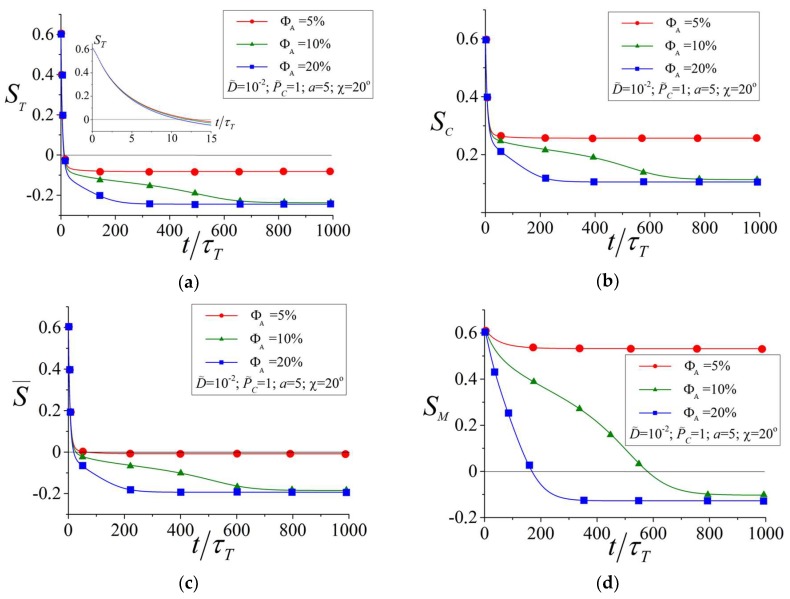
Order parameters for different orienting moieties: for the trans-(**a**) and cis-(**b**) isomers of azobenzenes, for all azobenzenes (**c**) and the mesogens (**d**) as functions of time at different volume fraction of azobenzenes.

Light-induced reorientation of azobenzenes caused by the angular selectivity of trans-cis photoisomerization results in the reorientation of the mesogens due to the mutual LC interactions and leads to network deformation. Changes of the orientational ordering in the two-component system and the network deformation depend first of all on the volume fraction $\Phi_{A}$ of the azobenzenes. Figure 3a–d shows the order parameters for different orienting moieties at different values of $\Phi_{A}$. Here, the averaged order parameter for all azobenzenes is defined as follows:

(25) $$\bar{S}=\frac{\Phi_{T}}{\Phi_{A}}S_{T}+\frac{\Phi_{C}}{\Phi_{A}}S_{C}$$

From Figure 3a–c, one can see that at short times, $t<10\tau_{T}$, the change of the order parameters for the azobenzenes and their isomers is almost independent of the amount of the azobenzenes and is defined by the characteristic time $\tau_{T}$, which depends only on the intensity of the light. Thus, the first stage of the photoisomerization kinetics, $t<10\tau_{T}$, is determined by the light-induced reorientation of the azobenzenes.

At long times, $t>10\tau_{T}$, the LC interactions between the azobenzenes and mesogens, the strength of which depends on the volume fractions $\Phi_{A}$ and $\Phi_{M}$, come into play. Therefore, kinetics of the light-induced reordering at $t>10\tau_{T}$ depends on the amount of azobenzenes. One can see from Figure 3a–d that an increase of $\Phi_{A}$ leads to a larger change of the order parameters for all components. This result illustrates a unique interplay between the components: a larger amount of azobenzenes leads to a larger change of the orientational order of the mesogens and a larger change of the orientational order of the mesogens leads back to a larger change of the orientational order of the azobenzenes due to the LC interactions between the components.

Interestingly, a small amount of azobenzenes ($\Phi_{A}=5\%$) is not enough to reorient the mesogens perpendicularly to the polarization direction **E**: the order parameter $S_{M}$ changes only slightly, remaining positive (Figure 3d). To reorient the mesogens perpendicularly to **E** with negative values of the order parameter $S_{M}<0$, a sufficiently large amount of azobenzenes ($\Phi_{A}\geq 10\%$) is needed. Similar results are observed also for the network deformation.

**Figure 4:**
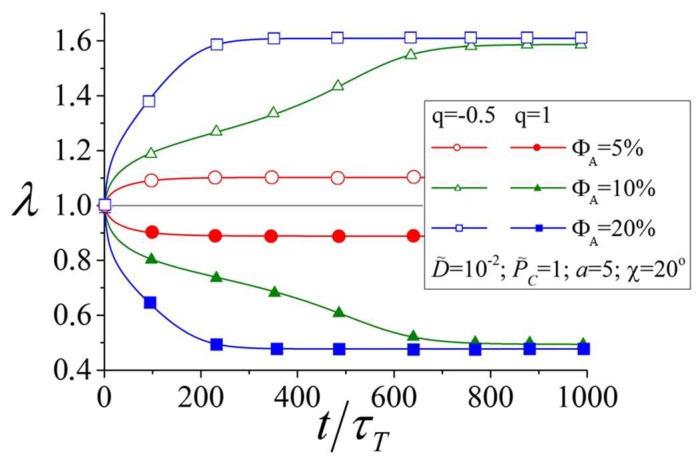
Elongation ratio as a function of time for networks with a different amount of the azobenzenes and with preferable orientation of azobenzenes and mesogens parallel ($\;q_{A}=q_{M}\equiv q=1$) or perpendicular $\;q_{A}=q_{M}\equiv q=-0.5$ to the main chains.

Figure 4 illustrates the elongation ratio as a function of time for the networks with the same values of the structural parameters as given in Figure 3a–d. For calculations of *λ*, we used Equations (1)–(5), in which the number fractions of azobenzenes and mesogens in polymer chains were defined as $N_{A}/N=\Phi_{A}$ and $N_{M}/N=\Phi_{M}$. One can see from Figure 4 that although the mesogens remain oriented preferably along the polarization vector **E** at $\Phi_{A}=5\%$ with $S_{M}>0$, the elongation ratio is determined by the total change of the order parameters according to Equations (1)–(5) and, thus, some network deformation is observed at $\Phi_{A}=5\%$. Larger network deformations take place at higher amounts of the azobenzenes with $\Phi_{A}\geq 10\%$ (see Figure 4). This tendency was observed in experiments: in Ref. [57], it was shown that an increase of the volume fraction of azobenzenes from 10% to 30% leads to a larger degree of network deformation under illumination with light. The bending deformation of azobenzene-containing LC networks was observed already at $\Phi_{A}=5\%$ [24].

Furthermore, one can see from Figure 4 that the direction of the network deformation, i.e., expansion (λ>1) or contraction (λ<1) along the polarization direction **E**, is determined by the orientation distribution of the azobenzenes and mesogens with respect to the main chains. Azobenzene-containing LC networks demonstrate expansion/contraction for the networks with preferable orientations of the azobenzenes and mesogens perpendicular (q=−0.5)/parallel (q=1) to the main chains, respectively. This result is confirmed by experiments [24] showing that a change of the orientation distribution of azobenzenes with respect to the main chains can invert the direction of network deformation.

Interestingly, the time dependences of order parameters and the elongation ratio at $\Phi_{A}\geq 10\%$ are characterized by an S-shaped form with several typical slopes, whereas these dependences at $\Phi_{A}=5\%$ have monotonically decreasing slopes. The S-shaped time dependences of light-induced deformation have been observed experimentally [23,24]. To understand the appearance of time dependences of order parameters and deformation of such a form, we analyzed the free energy for mesogens as a function of time, similarly to Doi and Edwards [51]. The free energy can be calculated as F=U−TΞ, where U is the internal energy and Ξ is the orientational entropy. The latter is related to the averaged logarithm of the orientational distribution function n(θ): Ξ=−k〈lnn(θ)〉. The part of the free energy $F_{M}$ (per mesogen), which depends on the order parameter of the mesogens $S_{M}$, can be obtained in the following form using Equations (12) and (22):

(26) $$\frac{F_{M}}{kT}=-\frac{1}{2}a_{MM}\Phi_{M}S_{M}^{2}-a_{TM}\Phi_{T}S_{T}S_{M}+m_{M}\frac{2S_{M}+1}{3}-\ln\left[\int_{}d\Omega \exp(m_{M}\cos^{2}\theta )\right].$$

Using the last equation, we calculated the free energy $F_{M}$ as a function of $S_{M}$ at different times, the parameters $\Phi_{T}$ and $S_{T}$ being taken from the solution of kinetic Equations (17)–(21).

**Figure 5:**
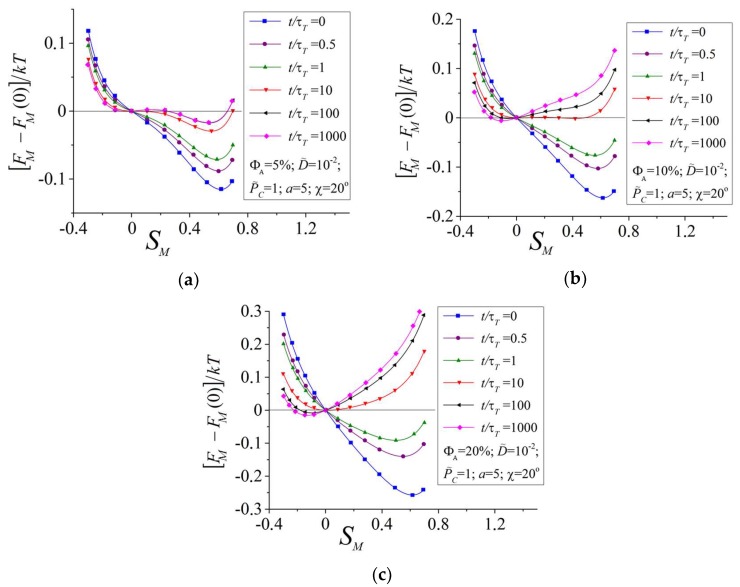
Free energy of mesogens $F_{M}$ as a function of the order parameter of mesogens $S_{M}$ at different times for two-component azobenzene-containing liquid crystalline (LC) networks with $\Phi_{A}=5\%$ (**a**), 10% (**b**), and 20% (**c**).

Figure 5 shows the free energy $F_{M}$ as a function of $S_{M}$ at different times for two-component networks with different volume fractions of azobenzenes $\Phi_{A}=5\%$ (a), 10% (b), and 20% (c). At t=0, the free energy $F_{M}$ has the only minimum at a positive value of the order parameter $S_{M}\approx 0.6$, which corresponds to the initial LC state (cf. with Figure 3d). With increasing time, the order parameter $S_{T}$ for the trans-isomers decreases due to the angular hole burning effect as presented in Figure 3a. This leads to the increase of the free energy $F_{M}$ in the region $S_{M}>0$ due to the contribution of the second term in Equation (26) for all $\Phi_{A}$. However, the stationary values of the free energy differ for $\Phi_{A}=5\%$ and for $\Phi_{A}\geq 10\%$.

One can see from Figure 5a that at $\Phi_{A}=5\%$ the free energy is almost unchangeable at $t>100\tau_{T}$ and demonstrates the global minimum at a positive $S_{M}\approx 0.55$ corresponding to the stationary state in Figure 3d. Thus, at a small amount of azobenzenes, the minimum of the free energy at $S_{M}>0$ cannot rise above the level $F_{M}\left(S_{M}=0\right)$ and the subsystem of mesogens remains in a state with positive values of the order parameter $S_{M}>0$.

At a sufficiently large amount of azobenzenes $\Phi_{A}\geq 10\%$, the minimum of the free energy at $S_{M}>0$ can reach the level $F_{M}\left(S_{M}=0\right)$ at $t\approx 10\tau_{T}$, and with a further increase of time the global minimum of the free energy appears at negative values of the order parameter $S_{M}<0$ (Figure 5b). The system chooses the state with a minimal value of the free energy and, thus, it transforms from a state with a positive value of $S_{M}$ to a state with a negative $S_{M}$. It should be noted that the speed of change of the order parameter $S_{M}$ is defined by the slope of the function $F_{M}\left(S_{M}\right)$ at the current value of $S_{M}$. One can see from Figure 5b that at $t\approx 10\tau_{T}$ the free energy for $\Phi_{A}=10\%$ has a very weak slope in the region between two metastable states with $S_{M}<0$ and $S_{M}>0$. This leads to the appearance of a specific inflection on the time dependences $S_{M}\left(t\right)$ (Figure 3d). Due to the LC interactions between azobenzenes and mesogens, such specific inflections appear also on the time dependences $S_{T,C}\left(t\right)$ and, as a consequence, on the time dependences $\bar{S}\left(t\right)$ and λ(t).

Thus, the S-shaped time dependences of the elongation ratio, which are observed also in experiments [23,24], can be due to the presence of two metastable states with different degrees of LC ordering. A slow transformation between these metastable states can lead to the appearance of specific inflections on the S-shaped time dependences of the elongation ratio λ(t).

### 3.2. Influence of the Orientational LC Interactions between Azobenzene Chromophores and Mesogens

**Figure 6:**
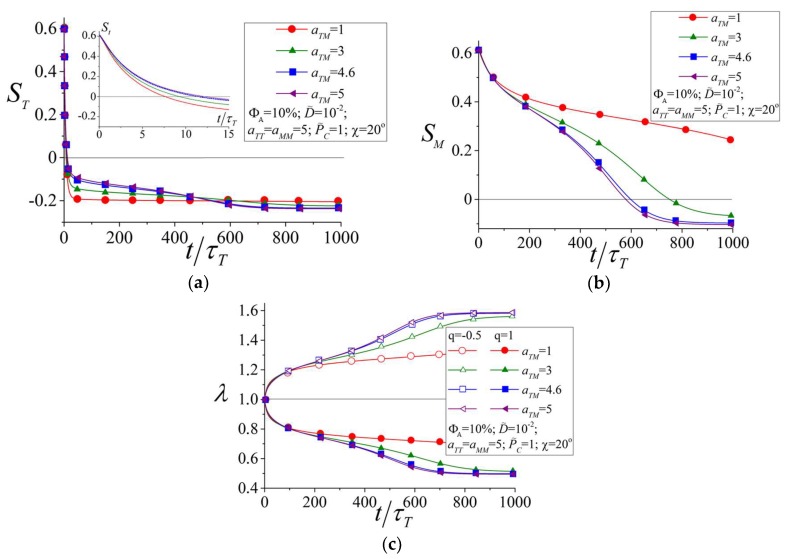
Order parameters for the trans-isomers of azobenzenes (**a**) and for mesogens (**b**) as well as the elongation ratio of the network (**c**) as functions of time at different strengths of the LC interactions between the azobenzenes and mesogens $\;a_{TM}$.

The strength of the LC interactions between azobenzenes and mesogens is determined by the parameter $a_{TM}$. Figure 6a–c show the time dependences of the order parameters $S_{T}$ (a) and $S_{M}$ (b) and the elongation ratio λ (c) at different values of $a_{TM}$ belonging to the regions $a_{TM}<a_{c}$ and $a_{TM}>a_{c}$. One can see that at very short times, $t<\tau_{T}$, the light-induced reordering and deformation are almost independent of strength of the LC interactions. As was mentioned in the previous section, the initial stage of the photoisomerization kinetics is defined by the hole burning effect and depends mostly on the intensity of the light.

At longer times, $t>\tau_{T}$, the influence of the mesogens on the light-induced reorientation of azobenzenes can be separated into two effects. First, the mesogens, which are oriented initially preferably along the polarization **E**||**n**, hinder reorientation of the azobenzenes perpendicularly to the light polarization due to the LC interactions. This leads to a slowing down of the reorientation kinetics of the azobenzenes at $\tau_{T}<t<100\tau_{T}$ with increasing strength of the LC interactions $a_{TM}$ (see Figure 6a). On the other hand, light-induced reorientation of azobenzenes results in the reorientation of the mesogens due to the LC interactions between the components. The stronger are the LC interactions, the stronger is the change of the order parameter of the mesogens $S_{M}$ (see Figure 6b). As a result, the stronger reorientation of the mesogens leads to the stronger back response of the mesogens on the azobenzenes and the azobenzenes are reoriented stronger with respect to the vector **E**. This is displayed in more negative values of the order parameter $S_{T}$ at long times, $t>500\tau_{T}$, with increasing strength of LC interactions (see Figure 6b).

Thus, an increase of the LC interactions between azobenzenes and mesogens results in stronger reorientation of both components perpendicular to the light polarization. This leads to larger light-induced deformations at higher strengths of the LC interactions as can be seen from Figure 6c. This result justifies the reasonability of using two-component networks with strong LC interactions to create materials which are able to produce light-induced deformations of large magnitudes. Such photodeformable materials based on LC polymer networks are widely reported in the literature [15,16,23,24,25,57,58,59,60,61,62,63].

### 3.3. Influence of the Viscosity of the Material

If the temperature approaches the glass transition temperature, $T_{g}$, from above, the viscosity of an amorphous material can increase dramatically [64]. In the region near $T_{g}$, a decrease of temperature by 10–20 K corresponds to an increase of the viscosity by 2–4 decades [64]. This is accompanied by significant slowing down of the molecular mobility, and the rotational diffusion coefficient D can decrease significantly at $T\to T_{g}$. Thus, near $T_{g}$ there exists a region of temperature in which the strengths of the LC interactions $a_{ij}=u_{ij}/kT$ change only slightly, whereas the rotational diffusion coefficients $D_{i}$ can change significantly. Therefore, it is interesting to investigate the effects of viscosity on the kinetics of light-induced ordering and deformation.

The rotational diffusion coefficient D of a rod-like particle is related to the viscosity, *η*, as follows [51]:

(27) $$D=3kT(\ln2p-0.5)/\pi \eta L^{3}$$

where L and p are the length and aspect ratio of the particle, respectively. At $T\to T_{g}$, the viscosity reaches the characteristic values $\eta \approx 10^{12}\;\mathrm{Pas}$ [60]. Using geometrical characteristics of the azobenzene chromophore L≈9 nm and p≈3, we found that its rotational diffusion coefficient can take the values $D>10^{-5\;}s$ if $\eta <10^{12}\;\mathrm{Pas}$. This corresponds to the region of the dimensionless parameter $\tilde{D}=D/P_{T}>10^{-4}$ at the characteristic intensity of light $I=0.1\;W/{\mathrm{cm}}^{2}$ used in experiments. Here, the probability of optical excitation $P_{T}=k_{TC}I$ was estimated using a typical value of the rate constant $k_{TC}\approx 0.4{\;\mathrm{cm}}^{2}/J$ [43]. The results presented in Figure 3, Figure 4, Figure 5 and Figure 6 were obtained for $\tilde{D}=10^{-2}$, which corresponds to the region 10–20 K above the glass transition temperature. In the present section, we shall consider the effects of viscosity while varying the parameter $\tilde{D}$ in the region $\tilde{D}>10^{-4}$.

**Figure 7:**
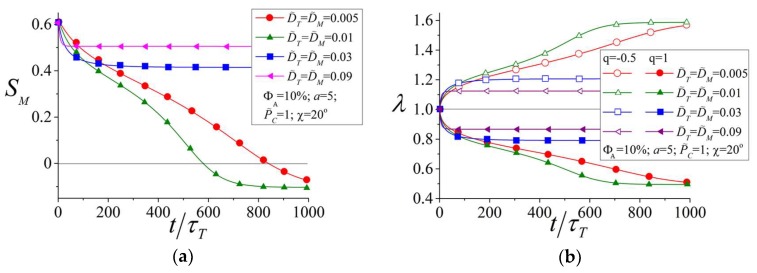
Order parameter for mesogens (**a**) and elongation ratio of the network (**b**) as functions of time at different reduced rotational diffusion coefficients ${\tilde{D}}_{T,M}$.

Figure 7 shows the order parameter for the mesogens (a) and the network elongation ratio (b) as a function of time at different values of the reduced rotational diffusion coefficients ${\tilde{D}}_{T,M}$. The values ${\tilde{D}}_{T}$ and ${\tilde{D}}_{M}$ for azobenzenes and mesogens were chosen to be identical. This case corresponds to those structures in which the mesogens have geometrical characteristics (lengths, aspect ratios, etc.) similar to the azobenzenes. Moreover, as was shown by Tiberio et al. [65], the rotational diffusion coefficients $D_{T,C}$ for trans- and cis isomers of azobenzenes are very close to each other. Thus, everywhere below we will use an approximation ${\tilde{D}}_{C}={\tilde{D}}_{T}$.

Two effects can be seen from Figure 7. First, a decrease of the rotational diffusion coefficients ${\tilde{D}}_{T,M}$ due to an increase of the viscosity leads to slowing down of the light-induced reordering and deformation in the region $t<100\tau_{T}$. This result of theory explains the tendency observed in many experiments: the characteristic time of photo-orientation and photodeformation increases significantly, from several seconds up to minutes and hours, with the decrease of temperature from the viscoelastic state above $T_{g}$ to the glassy state below $T_{g}$ [15,16,23,24,25,57,58,59,60,61,62,63].

The second effect is related to the stationary behavior of light-induced ordering and deformation at long times. From the kinetic Equations (17) and (18) it can be seen that the stationary state at long times when $\partial S_{T,C}/\partial t=0$ is defined by a balance between the optical terms proportional to $P_{T,C}$ and the diffusion terms, which are determined by $D_{T,C}$. Thus, more intensive Brownian motions prevent light-induced ordering and deformation. Therefore, an increase of ${\tilde{D}}_{T,M}$ results in a smaller change of the order parameter $S_{m}$ and in smaller light-induced deformations. The smaller light-induced deformation result has been confirmed by the experiment [66] showing that an increase of temperature prevents inscription of the surface relief gratings on azobenzene polymer films at a constant intensity of light. Thus, although an increase of temperature can speed up the process of light-induced deformation, it can decrease the magnitude of deformation. To reach an optimal regime of rapid and significant photodeformations, intermediate temperatures should be chosen.

### 3.4. Influence of the Wavelength of Light

One of the factors that influences the photomechanical behavior is the wavelength of light. Its variation at a fixed light intensity changes the ratio between the probabilities of trans-cis, $P_{T}$, and cis-trans, $P_{C}$, optical excitations $P_{C}/P_{T}>1$ for visible light, whereas $P_{C}/P_{T}<1$ for UV light [43]. In the present section, varying the ratio ${\tilde{P}}_{C}=P_{C}/P_{T}$, we study the influence of the wavelength of light on the light-induced ordering and deformation of azobenzene-containing LC polymer networks.

**Figure 8:**
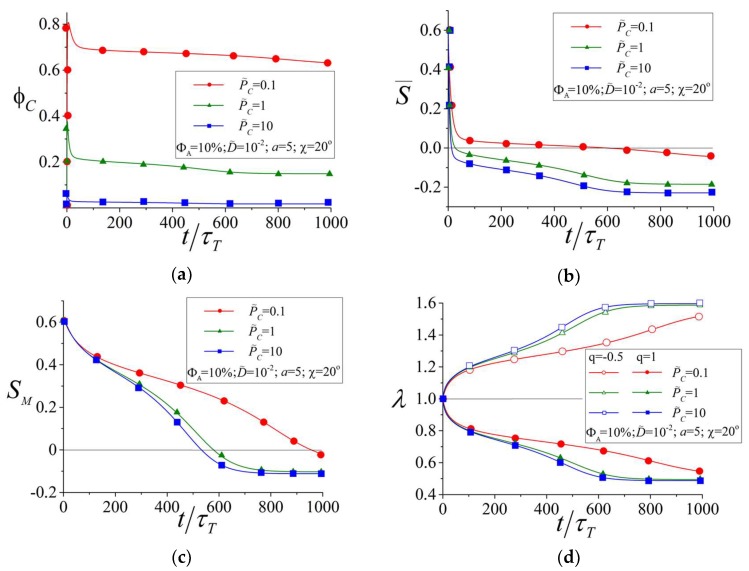
Number fraction of cis-isomers $\varphi_{C}$ (**a**), the order parameters $\bar{S}$ for all azobenzenes (**b**) and $S_{M}$ for the mesogens (**c**), and the network elongation ratio λ (**d**) as functions of time at different values of ${\tilde{P}}_{C}=P_{C}/P_{T}$.

Figure 8 shows the time dependences of the number fraction of cis-isomers $\varphi_{C}=\Phi_{C}/\Phi_{A}$ (a), the order parameter for all azobenzenes (b), the order parameter of the mesogens (c), and the elongation ratio of the network (d) at different values of ${\tilde{P}}_{C}$. of cis-isomers $\varphi_{C}$ first increases from zero due to the predominant trans-cis isomerization at $t<\tau_{T}$. This can be understood by looking at the kinetic Equations (17)–(20), where the derivatives ${\left(\partial S_{T,C}/\partial t\right)}_{t=0}$, which determine the photoisomerization kinetics in the initial stage, are solely defined by the probability of trans-cis isomerization $P_{T}$, being independent of the probability of cis-trans isomerization $P_{C}$, since cis-trans isomerization $P_{C}$ appears in the combination $P_{C}\Phi_{C}$ and $\Phi_{C}=0$ at t=0. In the next stage, $t>\tau_{T}$, the back cis-trans isomerization process comes into play and $\varphi_{C}$ decreases as a function of time, showing a maximum around $t\approx \tau_{T}$ (see Figure 8a).

The stationary value of $\varphi_{C}$ at t→∞ depends on the ratio ${\tilde{P}}_{C}$. For instance, at ${\tilde{P}}_{C}=0.1$, which corresponds to UV light, the number fraction of cis-isomers can reach the values $\varphi_{C}\approx 60-80\%$ in agreement with the experimental data [43]. At the same time, at ${\tilde{P}}_{C}=10$, which corresponds to visible light, the number fraction of cis-isomers is rather small, $\varphi_{C}\approx 5-10\%$, also in agreement with the experiment [43]. Since the cis-isomers have a bent form, they reduce the intensity of the LC interactions. As a result, the stationary value of the order parameter for all azobenzenes is $\bar{S}\approx 0$ at ${\tilde{P}}_{C}=0.1$ (see Figure 8b), whereas $\bar{S}\approx -0.2$ at ${\tilde{P}}_{C}=10$. This leads to a larger change of the order parameter for mesogens $S_{M}$ and to a larger degree of deformation λ at ${\tilde{P}}_{C}=10$ as compared to the values of $S_{M}$ and λ at ${\tilde{P}}_{C}=0.1$ (see Figure 8c,d).

Thus, we expect that visible light with the polarization **E**||**n** can induce larger network deformations as compared to the deformations caused by irradiation with UV light. This is explained by a large amount of rod-like trans-isomers under illumination with visible light and by reorientation of the trans-isomers perpendicular to the polarization direction **E**, which provides an additional contribution to the deformation as compared to a simple destruction of the LC state by bent cis-isomers under UV irradiation.

## 4. Conclusions

Kinetics of the light-induced ordering and deformation in two-component polymer networks consisting of azobenzene chromophores and optically inert mesogens have been studied using the time-dependent equations of photoisomerization. The angular selectivity of trans-cis isomerization of azobenzenes as well as orientational LC interactions between the rod-like trans-isomers of azobenzenes and mesogens are taken into account. Effects of dilution of the LC state by bent cis-isomers of azobenzenes are considered. Photomechanical behavior has been studied for a geometry of light illumination in which the polarization vector of light is parallel to the initial director of the LC network. As was predicted in Ref. [37], this geometry provides the largest degree of light-induced deformation.

Light-induced reorientation of azobenzenes caused by the angular selectivity of photoisomerization results in a change of the orientational order of mesogens due to the LC interactions between the components. This can lead to the appearance of two metastable orientational states corresponding to two local minima of the free energy with different values of the order parameter. A slow transition between the two metastable states manifests in the appearance of S-shaped time dependences of the degree of deformation that were observed in a number of experiments [23,24].

We have shown that the kinetics of light-induced ordering and deformation of azobenzene-containing LC networks depends on the intensity and wavelength of light as well as on the mechanical characteristics of the network and its chemical structure. For example, an increase of the intensity of light accelerates the kinetics of light-induced reorientation. This fact is utilized in the experimental technique [11] for rapid inscription (~10 ms) of the director field in an alignment layer using a sufficiently high intensity of light ~40 W/cm^2^. A change of the wavelength from UV to visible light increases the fraction of rod-like trans-isomers of azobenzenes and leads to additional ordering of azobenzenes and mesogens perpendicular to the light polarization and to a larger degree of deformation. Thus, at the same level of light absorption, we can expect larger deformations under illumination with visible light as compared to the deformations under UV light, whose main effect is a destruction of the LC state by the bent cis-isomers.

An increase of the amount of azobenzenes in a polymer network increases the magnitude of light-induced deformation in agreement with experiments [57]. Depending on the orientation distribution of azobenzenes and mesogens with respect to the main chains, the azobenzene LC networks can demonstrate either expansion or contraction with respect to the light polarization. This result is confirmed by experiments [24] showing that a change of the orientation distribution of azobenzenes with respect to the main chains can invert the direction of network deformation. An increase of the strength of the LC interactions between azobenzenes and mesogens results in larger light-induced deformations. This result justifies the reasonability of using two-component networks with strong LC interactions to create materials which are able to produce light-induced deformations of large magnitudes [15,16,23,24,25,57,58,59,60,61,62,63]. An increase of temperature from the region near $T_{g}$ decreases dramatically the viscosity of the material and accelerates the process of light-induced deformation. However, due to more intensive random Brownian motions, it can decrease the magnitude of directional deformation in accordance with the experiment [66] showing that an increase of temperature prevents the inscription of surface relief gratings. To reach an optimal regime for rapid photodeformation of a high magnitude, intermediate temperatures should be chosen.

To conclude, the established structure-property relationships are in agreement with a number of experimental data and can be useful for the creation of sophisticated photodeformable materials with target technologically important properties.
